# Postoperative complications in cochlear implant surgery and their possible risk factors

**DOI:** 10.1016/j.bjorl.2024.101428

**Published:** 2024-03-25

**Authors:** Vanessa Ribeiro Orlando, Oswaldo Laércio Mendonça Cruz

**Affiliations:** a Hospital Santa Casa de Belo Horizonte, Belo Horizonte, MG, Brazil; b Universidade Federal de São Paulo (UNIFESP), Departamento de Otorrinolaringologia e Cirurgia de Cabeça e Pescoço, São Paulo, SP, Brazil; c Universidade Federal de São Paulo (UNIFESP), Escola Paulista de Medicina, São Paulo, SP, Brazil; d Universidade de São Paulo (USP), São Paulo, SP, Brazil; e Universidade Federal de São Paulo (UNIFESP), São Paulo, SP, Brazil; f Universidade Federal de São Paulo (UNIFESP), Hospital São Paulo (HSP), Centro do Deficiente Auditivo, São Paulo, SP, Brazil

**Keywords:** Hearing loss, Cochlear implantation, Risk factors, Device failure, Reoperation

## Abstract

**Objective:**

This is a retrospective analysis of the major and minor complications of cochlear implants, as well as the Risk Factors (RF) involved.

**Methods:**

We analyzed the medical records of patients submitted to cochlear implants at public University from 2006 to July 2019, and list here the major and minor complications found, and their risk factors.

**Results:**

There were 193 ears, 100 (51.3%) from females and 93 (48.2%) from males, with a mean age of 23.63 years. In 54 of them (28%), there were alterations seen in the Temporal Bone CT scan, and 44 (22.8%) in the brain MRI. There were 158 (81.9%) insertions performed; 127 (65.8%) of them through the round window. There were 78 complications: 19 (9.8%) major and 56 (29%) minor complications. Among the major complications, there were 3 (1.6%) Surgical Site infections (SS); 5 (2.6%) hematomas/seromas; 5 (2.6%) electrode extrusion; 5 (2.6%) device faults; 1 (0.5%) wrong path. Among the minor complications, there were 6 (3.1%) Acute Otitis Media (AOM); 9 (4.7%) SS infections; 4 (2.1%) facial paresis; 17 (8.8%) vertigos; 9 (4.7%) with tinnitus. The most important RF was age. Patients younger than 2.5 years had more major complications: SS infection (*p* = 0.018) and electrode extrusion (*p* = 0.017). There was a higher rate of vertigo in adults (*p* = 0.003), and it was more often associated with comorbidities (*p* = 0.008). The insertion route, the presence of changes in CT and MRI and the CI brand used did not impact the number of complications.

**Conclusion:**

Among the minor complications, those involving the vestibular system were the most common, especially in adults with comorbidities. Regarding major complications, there was an emphasis on SS infections, hematomas, seromas, electrode extrusion, especially in children under two years of age. There were implanted device faults (2.6%), with none of the brands evaluated standing out.

## Highlights

• Minor complications related with vestibular system were more common.
• Major complications: changes of wound, device failure and electrode extrusion.
• Children with a mean age of 19.3-months are more susceptible to infections.
• There was no correlation between advanced age and higher complication rates.
• Age should not be an exclusion factor.

## Introduction

Cochlear Implants (CI) are electronic devices for hearing rehabilitation of individuals with severe or profound sensorineural hearing loss. There are different models and techniques that can be used to improve results. Several studies have reported low rates of postoperative complications [1], [2], [3]. Nevertheless, complications may ensue, and their relevance is that in addition to the inconvenience for the patient, they impact on healthcare system financials [3], [4].

There are several classifications for these CI complications. One of the most used is Cohen's 1995 classification, which divides them into minor and major complications. The minor ones are those that resolve spontaneously or with conservative treatment, without the need for surgical intervention. While the major complications require hospitalization, surgery and/or explant [4].

In contrast to reports of these CI complications, there are some, but few studies related to risk factors [5]. Thus, the goal of this study was to retrospectively analyze CI major and minor complications and assess risk factors behind their occurrence.

## Methods

This was a retrospective study, level of evidence Step 3, carried out from the medical records of patients submitted to cochlear implantation from a Federal University of São Paulo (UNIFESP) from 2006 to July 2019; and it was approved by the Research Ethics Committee (CEP), CAAE: 24090819900005505. This institution receives and treats cases of greater clinical and surgical complexity and trains new otorhinolaryngologists, who actively participate in cochlear implant procedures.

For this purpose, the following epidemiological data were evaluated: age, gender, hearing loss etiology, comorbidities, radiological alterations shown in the Magnetic Resonance Imaging of the inner ear (MRI) and Computed Tomography of the temporal bones (CT). In addition, we also assessed the brand of the implant used and the way in which the electrodes were inserted during the surgical procedure.

Thus, the frequency and type of postoperative complications were evaluated within the period of 1–10 years after the procedure. Complication severity was divided into major and minor according to Cohen.

To correlate with complications, the following risk factors were analysed: age, consanguinity, meningitis, inner ear malformations, autoimmune diseases, Meniere’s disease, temporal bone fracture, acute otitis media, chronic otitis media, changes in the CT and/or MRI, comorbidities, route of CI electrodes insertion and CI brand.

Patients with incomplete medical records and those without adequate clinical follow-up were taken off the study. Those who had a minimum follow-up of 1-year and a maximum of 10-years after the surgical procedure remained, with a mean follow up time of 5.4-years (SD = 3).

For statistical analysis, we used the SPSS for Windows 20.0 software (SPSS, Inc., IL, USA). The quantitative variables were expressed as mean and standard deviation. The Chi-Square test, Contingency Coefficient and the *t*-test were used in the analyses considering a *p* < 0.05 as significant.

## Results

**Fig. 1:**
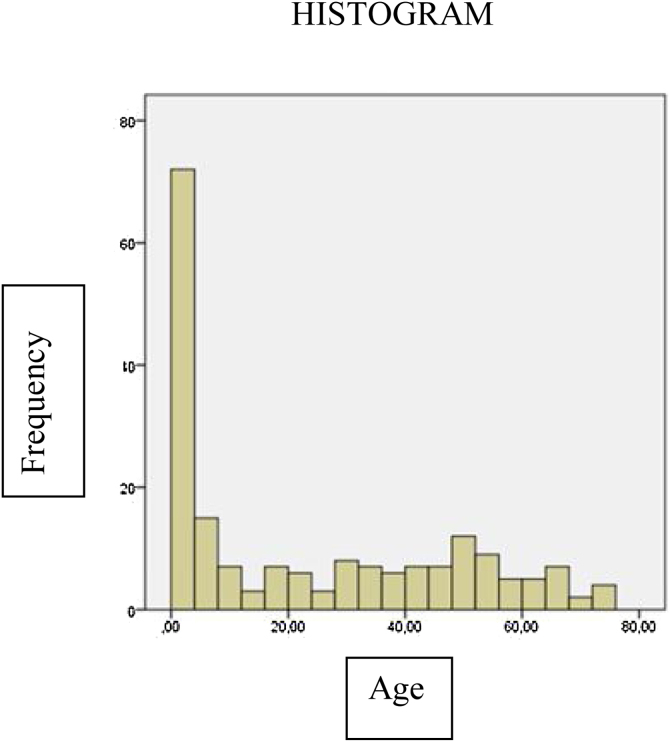
Patients according to age at the time of the surgical procedure.

We had 193 implanted ears: 100 (51.3%) from females and 93 (48.2%) from males. The mean age at the time of surgery was 23.63 (SD = 23.26) years, the youngest being 1-year old and the oldest 74-years of age. Of the total number of patients, 54.2% were under 20 years of age (Fig. 1).

Factors associated with the hearing loss etiology were: 44 patients (22.8%) had a family history of hearing loss; 27 had had (14%) meningitis; in 24 (12.4%) the cause was not found; 19 (9.8%) had been premature; 16 (8.3%) had been to a neonatal ICU; 15 (9.1%) had ototoxicity; 13 (6.7%) had congenital infections; 12 had other infections (6.2%); 11 (5.7%) had malformations; 11 (5.7%) had otitis media; 10 (5.2%) had neonatal jaundice; 9 (4.7%) had consanguinity; 9 (4.7%) neonatal infections; 8 (4.1%) had neonatal asphyxia; 8 (4.1%) had autoimmunity issues; 8 (4.1%) had some auditory neuropathy; 6 (3.1%) had otosclerosis; 4 (2.1%) had Meniere; 4 (2.1%) had trauma.

Concerning their CT scan, 54 (28%) patients had changes; 20 had serous otitis media; 8 had enlarged vestibular aqueduct syndrome; 7 had ossifying labyrinthitis; 6 had chronic otitis media; 3 had retrofenestral otosclerosis; 2 had Mondini-type incomplete bipartitions; 2 had temporal bone fractures, 2 had vestibular malformations; 1 had a granuloma; 1 had a narrow internal auditory canal and 1 had a cholesterol granuloma.

MRI showed 44 (22.8%) alterations: 20 serous otitis media; 12 intracochlear fibrosis; 4 chronic otitis media; 2 Mondini; 2 enlarged vestibular aqueduct syndrome; 1 cytomegalovirus; 1 cholesterol granuloma; 1 acoustic neuroma with bilateral hearing loss and 1 vestibular malformation.

The four brands of CI available on the market were used (Table 1).

**Table 1:** Cochlear implants used in surgical procedures according to brand.

Brand	Frequency	Percentage
Cochlear	93	48.2
Med El	50	25.9
Ad.Bionics	27	14.0
OTICON	20	10.4
Neurelec	3	1.6
Total	193	100.0

As for the most significant and prevalent comorbidities, we found 21 patients (10.9%) with systemic arterial hypertension; 12 patients (6%) with diabetes mellitus; 5 patients (1%) with severe heart diseases; 5 (2.6%) with hypothyroidism; 5 (2.6%) had depression; 4 (2%) had dyslipidemia; 4 (2%) patients had difficult-to-control asthma; 4 (2%) had chronic renal failure; 3 (1.5%) patients were smokers; 4 (2%) had severe asthma; 1 (0.6%) patient suffered from acquired human immunodeficiency syndrome; 4 (2%) patients had cognitive impairment, and 1 (0.5%) had idiopathic thrombocytopenic purpura.

Among the 193 surgeries assessed, 158 (81.9%) electrodes were completely inserted; 35 (18.1%) were partially inserted. The main insertion route was the round window, performed in 127 (65.8%) patients; and in 66 (34.2%) of them the insertion occurred through a cochleostomy.

### Complications

There were 78 complications, including 19 major and 56 minor complications. Thus representing 9.8% of all surgeries for major complications and 29% for minor complications.

As major complications, 3 (1.6%) were surgical wound infections; 5 (2.6%) patients developed hematomas/seromas; 5 (2.6%) had electrode extrusion; in 5 (2.6%) patients the device failed; and there was 1 (0.5%) electrode improper placement (Table 2).

**Table 2:** There were 19 major complications, corresponding to 9.8% of the total surgeries evaluated in the study.

Major complications	Yes		Total
	n	%	n
Infection	3	1.6	193
Mastoiditis	0	0.0	193
Hematoma/seroma	5	2.6	193
Electrode extrusion	5	2.6	193
Device failure	5	2.6	193
CSF gusher	0	0.0	193
Electrode misplacement	1	0.5	193
Total	19	9.8	193

The 3 surgical wound infections occurred within the first 7 postoperative days in two cases, and within 30 days in the third case. The mean age of the patients was 19.3 months. Extrusion occurred in 5 patients, with a mean age of 1.6 years and an average of 9.5 months (SD = 2.5) after the procedure, there was a longer extrusion time, 60 months, in one of the patients because of a chronic cholesteatomatous otitis media and consequent extrusion.

Five patients had hematoma/seroma, among them, 3 had the complication by 7 days after surgery, one of the patients had seroma due to trauma 2 years after the procedure, requiring drainage; and one of them evolved with repeated seromas after 7 months of surgery, evolving to an explant.

As minor complications, there were 6 (3.1%) AOM; among them, three patients already had a history of otitis media; two had had a ventilation tube at the time of surgery and the third had a history of chronic otitis media. Of these AOMs, 3 occurred before the 10th POD, 1 on the 30th POD and two had it 6 months after the surgery.

There were 9 (4.7%) surgical wound infections, with superficial skin dehiscence, without an internal component extrusion. Of these infections, 6 occurred in the first postoperative week and the other 3 at the 30th POD.

Four patients had changes in facial mimicry (2.1%), with complete regression in 3 patients and 1 of them remained with House Brackmann (HB) grade 4 paresis. Two (1.0%) patients complained of extra auditory sensation with spasms in the facial muscles.

As for vestibular symptoms, vertigo occurred in 17 (8.8%) patients, it was not disabling and spontaneously regressed, requiring only symptomatic control. We had 9 (4.7%) patients complaining of tinnitus, and in 8 of them the symptoms of vertigo and tinnitus were concomitant (Table 3).

**Table 3:** There were 56 minor complications, corresponding to 29% of the surgeries in this study.

Minor complications	Yes		Total
	n	%	
AOM	6	3.1	193
Mastoiditis	0	0.0	193
Surgical wound infection	9	4.7	193
Facial paralysis	4	2.1	193
Extra-auditory sensation	2	1.0	193
Anesthetic complications	0	0.0	193
Vertigo	17	8.8	193
Tinnitus	9	4.7	193
External component failure	5	2.6	193
Skin rash	4	2.1	193
Total	56	29%	193

### Risk factors for the complications in the study

The most important factor was the age group at which the surgery was performed, with age lower than 2.5 years being associated with a major complication such as surgical wound infection (Contingency Coefficient = 0.242 e *p* = 0.018). Just as there was an association with electrode extrusion in this same age group (Contingency Coefficient = 0.242 e *p* = 0.017) a major complication.

Also age-related, we found a higher frequency of vestibular symptoms among the adults, there was a direct correlation with minor complications such as vertigo (Contingency Coefficient = 0.276 e *p* = 0.003), especially after 20 years of age. Children between 2.5 and 4 years old had a negative association with vertigo.

There was also an association between age and the occurrence of anomalous stimulus to the facial nerve among the minor complications (Contingency Coefficient = 0.232 e *p* = 0.03), especially in the age group from 20 to 39 years.

Regarding comorbidities, there was an association between the presence of comorbidities and the symptom of vertigo among the minor complications (Contingency Coefficient = 0.196 e *p* = 0.008). The comorbidities directly related to vertigo were hypertension (5 patients); depression (4 patients); diabetes (3 patients); and renal failure (2 patients).

There was a significant association between a past of AOM or COM and vertigo among the minor complications (Contingency Coefficient = 0.232 e *p* = 0.009).

The surgical insertion route of the electrode did not alter the expected CI result; thus, there was no correlation between complications seen in the study and the insertion route: cochleostomy or round window. Likewise, there was no greater number of complications in patients with alterations in the image exams: CT scan of the temporal bones and MRI of the inner ear, nor in relation to the brand or type of implant used in the procedure.

## Discussion

The wide indications for CI and its effectiveness in auditory rehabilitation have contributed to a significant increase in the number of implanted patients worldwide. However, as it has been indicated in increasingly younger patients, and also in the elderly, the concerns with complications have been the object of particular interest [7].

Therefore, the importance of good training and surgical skills on the part of the surgeon who performs the cochlear implant, avoiding complications related to the surgical technique. However, good technique alone will not abolish them. Therefore, the importance of evaluating related risk factors, trying to predict and prevent some of these complications.

CI manufacturers have been making efforts to minimize the incidence of device failure, which has contributed to the reduction of this complication. CI surgery is becoming increasingly safe thanks to this dynamic process in which implant centers and manufacturers work together to improve this modality of auditory rehabilitation. Thus, there is a need for cochlear implant centers to evaluate their results in their entire context, from the preoperative period to the postoperative outcome, with the aim of developing protocols aimed at reducing the risks of minor complications and, mainly, major ones.

Major complications, in this study, accounted for 10.4% of all surgeries, with good response to surgical clinical management, with no fatal outcome. The most common were related to the surgical wound with 3 (1.6%) surgical wound infections, 5 (2.6%) hematoma//seroma. Followed by 5 (2.6%) device failures and 5 (2.6%) electrode extrusions.

The rate of major complications from this study (9.8%) is close to the upper limit of that described in similar studies, which rates range from 3% to 13.7% [4]. Of these, the most common were also complications related to the surgical wound and device failures (1.84%–2.3%) [6].

There was a higher number of minor complications in this study, corresponding to 29%, compared to reports in the literature (11.8%) [7], which can be justified by the fact that our clinic receives and operates on cases of greater clinical and surgical complexity, in addition to training new ear surgeons, who actively participate in cochlear implant procedures.

The follow-up time of the patients was also long, considering that complications of patients with a minimum follow-up of 12 months after surgery and a maximum of 10 years were included, with a mean follow-up time of 5.4 years (SD = 3). Furthermore, there is no standardization for this type of complication in the literature and, in our study, we chose to include any and all complaints or disorders reported by the patients.

Four patients had changes in facial mimicry (2.1%), all of them with partial degree and with complete regression in 3 patients; one of the patients had a sequela and remained with the paresis, Grade 4 of HB. In the literature, the rate of facial paralysis ranges from 0.42% to 3.5% [2], [6], [8].

Two (1%) patients complained of extra auditory sensation with spasms in the facial muscles, with no risk factor identified. There was a higher prevalence of this complaint among between 20 and 39 years of age. None of the other evaluated risk factors was directly correlated with this fact, including the analysis of the 4 CI brands available on the market.

Risk factors associated with surgical wound infections include comorbidities such as Diabetes, advanced age, surgery duration and complexity. However, a chronic health condition as well as a history of chronic ear disease have been suggested, but not statistically proven, as risk factors for postoperative infections [5]. Thus, we evaluated the risk factors according to each complication reported.

As already reported in other studies [2], [4], [5], the lowest age range and the occurrence of surgical wound infections – a major complication ‒ was statistically significant (*p* = 0.018); and the mean age of patients with infection was 19.3 months (SD = 8).

This suggests a greater susceptibility to the development of postoperative infections in this age group, which may occur due to immune system immaturity. It can also be explained by a smaller head size and lower skin and subcutaneous tissue thickness. Device size is identical for both adults and children; therefore, a higher number of complications can be anticipated in children [2], [4], [5].

Infection is a worrisome complication, since the progression of the condition may result in the need to remove the implant, increasing the patient's morbidity and generating high costs for public coffers [4], [5].

There was also a statistically significant correlation between younger age and major complications, such as electrode extrusion (*p* = 0.017), with a mean age of patients who presented extrusion at 19.3 months of postop (SD = 10.1).

This can also be justified by the same aforementioned reasons, associated with cranial bone growth, especially the temporal/mastoid bones, and the greater risk of trauma to the head in this age group [4].

Among minor complications, the most frequent were those related to the vestibular system, which is in synch with other publications [2]. The majority regressed spontaneously, and in whom we just used symptomatic medication and watchful waiting.

There was a significant association between a history of AOM or COM and vertigo, in minor complications (*p* = 0.009). There was also a correlation between the highest age range and minor complications such as vertigo (*p* = 0.003), with the mean age of patients being 45.4 years (SD = 17) ‒ which was expected ‒ since it is difficult to assess this type of occurrence in the pediatric population, which is why we had a negative association between vertigo and patients aged 2.5–4 years.

The presence of comorbidities is directly related to minor complications such as vertigo (*p* = 0.008). The most frequent comorbidities in the face of vertigo were arterial hypertension, diabetes, renal failure and depression. Maybe because of the greater sensitivity of the cochleo-vestibular system in this group, due to metabolic and microvascular alterations caused by aging or previous disorders in the middle ear.

Considering that the vestibular system may not develop hypofunction to the same degree as the auditory system/cochlea, thus clinically expressing the surgery aggression (hydro-pressure imbalance and inflammation).

There was no increase in the number of complications according to the way in which the electrode was inserted, nor according to the brand of the equipment used.

There was no correlation between advanced age and higher complication rates. Elderly patients had a good response to the CI, and therefore age should not be an exclusion factor for these patients when considering this type of resource [2].

Here was no case of post-surgical meningitis, which has been described in other studies, attributed to pneumococcal vaccination. In our cases, there were no deaths or internal unit migration.

CI manufacturing companies should also strive to minimize the incidence of device failure, for they increase morbidity in these cases, working with and encouraging studies in large cochlear implant centers.

CI surgery is an established procedure, and the identification of risk factors can help predict and reduce even more the number of surgical complications, so it is important to work and encourage studies in large cochlear implant centers, and multicentric studies will help better establish such factors.

## Conclusion

Among the minor complications in this study, those related to the vestibular system were the most common, occurring mainly in patients over 42 years of age and with comorbidities such as systemic arterial hypertension, diabetes mellitus, chronic renal failure and depression. Regarding major complications, those related to the surgical wound, such as infection, hematoma, seroma, electrode extrusion, were highlighted, which were more frequent in the pediatric age group, especially in children under two years of age. Also noteworthy is implanted device failure of 2.6%, without picking any of the brands in the study.

## Funding

This work was financed by the authors themselves and there are no conflicts of interest.

## Conflicts of interest

The authors declare no conflicts of interest.
